# Supplementary material for: Complement C3-Deficiency-Induced Constipation in FVB/N-C3em1Hlee/Korl Knockout Mice Was Significantly Relieved by Uridine and Liriope platyphylla L. Extracts
Source: Int J Mol Sci. 2023 Oct 30;24(21):15757. doi: 10.3390/ijms242115757 (PMC10649790; doi:10.3390/ijms242115757)
Supplement: Supplementary file 1 [file ijms-24-15757-s001.zip › ijms-2595511-supplementary.pdf]

International Journal of *Molecular Sciences*

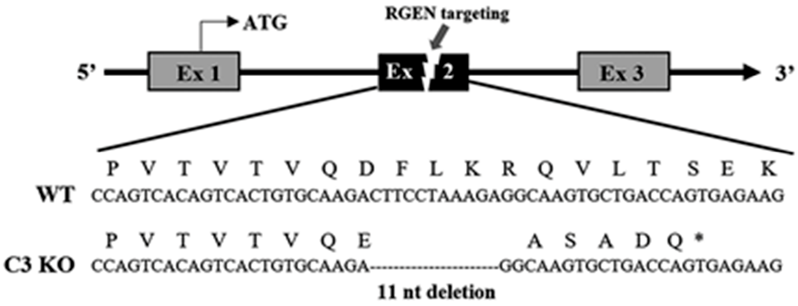

(**a**)

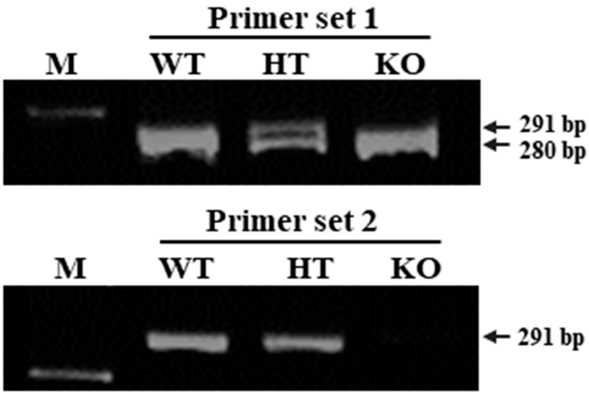

(**b**)

Supplement Figure S1. Targeting strategy of the C3 gene and identification of C3 KO mice. (a) The 11 nucleotides were deleted in exon 2 of the C3 gene to generate the premature form of the mutant C3 protein. (b) DNA-PCR typing of tail DNA. After amplification of PCR, the levels of the two products (291 and 280 bp) were quantified on 1% agarose gel. Abbreviations: WT, Wild type; C3 KO, Complement C3 knockout; HT, Heterogenous type; Ex, Exon.
